# *Notes from the Field*: Tetanus in an Unvaccinated Man from Mexico — Oregon, 2022

**DOI:** 10.15585/mmwr.mm7224a5

**Published:** 2023-06-16

**Authors:** Corey Pierce, Angela Holly Villamagna, Paul Cieslak, Juventila Liko

**Affiliations:** ^1^Public Health Division, Oregon Health Authority; ^2^Oregon Health & Science University, Portland, Oregon.

During June 2022, a non–English-speaking, Mexican-born male construction worker aged 42 years was evaluated at an Oregon emergency department (ED) complaining of 2 days of difficulty opening his mouth and pain in his back, arms, and neck. After receiving intravenous (IV) fluids and diazepam, he improved and was discharged. The following day, he visited a different ED with worsening symptoms, but again was discharged following administration of IV fluids and diazepam. He returned hours later with trismus and diffuse body spasms at which time a clinical diagnosis of tetanus was made. Immunization history was not documented during the first two ED visits. During the third ED visit, his family reported he had recently stepped on a nail at work and that he had no known history of tetanus immunization.

He was admitted to the hospital where he received IV metronidazole, tetanus immune globulin, tetanus toxoid, reduced diphtheria toxoid, and acellular pertussis vaccine (Tdap). Shortly thereafter, he experienced respiratory distress and was intubated. He was transferred to the intensive care unit (ICU) at a larger hospital, where a punctate callus was noted on the sole of the right foot. A tracheostomy was placed on day 13. He remained in the ICU through day 45. The tracheostomy was removed on day 48. At the time of discharge (day 50), he could speak, eat, drink, and walk short distances.

Tetanus is caused by a neurotoxin expressed by *Clostridium tetani*, an anaerobic, spore-forming gram-positive bacterium commonly found in soil and introduced through open wounds. Often life-threatening, tetanus can require months of medical care before recovery. During 2017, the cost for an unvaccinated pediatric patient in Oregon hospitalized with tetanus for 57 days exceeded $800,000 *(1)*. Tetanus is preventable through a primary 3-dose diphtheria and tetanus toxoids and acellular pertussis (DTaP) vaccination series at 2, 4, and 6 months, with booster doses at ages 15–18 months and 4–6 years. Persons aged 11–18 years should receive a single booster dose of Tdap, preferably during a preventive care visit at age 11–12 years. To ensure continued protection against tetanus and diphtheria, booster doses of Td or Tdap should be administered every 10 years throughout life. CDC recommends that adults with no history of vaccination against tetanus receive tetanus toxoid–containing vaccines recommended by the Advisory Committee on Immunization Practices.^*^ Migrant workers might be at increased risk for tetanus: their occupational injury risk has been shown to be twice that of U.S.-born workers *(2)*.

Health care providers should be on alert for reemerging vaccine-preventable diseases as a result of declines in vaccination coverage attributable to the COVID-19 pandemic.^†^ Providers should also be aware that migrant populations often have lower vaccination coverage rates and suffer higher rates of vaccine-preventable diseases than do nonmigrants (3). In the United States, vaccination coverage is significantly lower among non- U.S-born than among U.S.-born adults *(4)*. Across the Americas, national vaccination schedules have largely reached parity during recent decades, ensuring high coverage among children and young adults; however, coverage among adults is lower. Mexico, the country representing the largest U.S. migrant population, lags behind the United States in immunization metrics: during 2022, 3-dose DTaP coverage among Mexican children aged 1 year was 74%, compared with 93% in the United States, and receipt of 2 measles-containing vaccine doses by the nationally recommended age was 78% in Mexico and 85% in the United States.^§^ Providers in the United States should remain particularly vigilant for vaccine-preventable diseases among non–U.S.-born patients and take every opportunity to administer recommended vaccines.

Ineffective communication between providers and patients can delay treatment. The patient described in this report did not have his immunization history documented until his third ED visit, and only after the diagnosis of tetanus was apparent. To mitigate language barriers, clinical care settings should provide interpreter services for non–English-speaking communities. Providers should be mindful that some Hispanic or Latino immigrants in the United States have limited health literacy, hindering quality of care *(5)*. To address issues of health literacy, providers should consider use of both written and oral formats, photonovelas (picture stories), and “teach-back” methods^¶^.

This preventable tetanus case highlights the importance of equitable communication practices in health care settings, vigilance for serious but rare vaccine-preventable diseases, early ascertainment of immunization history, and awareness of possible lower vaccination coverage among migrant populations. Persons lacking verified immunization should be offered vaccination expeditiously.

## Footnotes

*
https://www.cdc.gov/mmwr/volumes/69/wr/mm6903a5.htm

†
https://www.who.int/news/item/15-07-2022-covid-19-pandemic-fuels-largest-continued-backslide-in-vaccinations-in-three-decades

§
https://www.who.int/publications/i/item/9789240051157

¶
https://doi.org/10.3912/OJIN.Vol14No03Man02
